# Lipid Metabolism in Cerebral Ischemia: From Pathogenesis to Therapy

**DOI:** 10.2174/011570159X389784250715064745

**Published:** 2025-07-23

**Authors:** Xinrong Wang, Rongjia Liu, Zhong Chen, Weiwei Hu, Lei Jiang

**Affiliations:** 1 Department of Pharmacology and Department of Pharmacy of The Second Affiliated Hospital, Key Laboratory of Medical Neurobiology of The Ministry of Health of China, School of Basic Medical Sciences, Zhejiang University School of Medicine, Hangzhou, 310058, China;; 2 Zhejiang Key Laboratory of Neuropsychopharmacology, Zhejiang Chinese Medical University, Hangzhou, 310053, China

**Keywords:** Lipid metabolism, cerebral ischemia, neuroinflammation, specialized pro-resolving mediators (SPMs), therapeutic targets, peroxisome proliferator-activated receptors (PPARs)

## Abstract

Cerebral ischemia, a leading global cause of death and disability, is marked by multifaceted pathological processes through dysregulation of lipid metabolism. This review examines the pivotal role of lipid metabolism in the pathogenesis of cerebral ischemia, with a particular emphasis on its dual function in neuroinflammation and neuroprotection. It delves into the mechanisms by which Arachidonic Acid (AA) metabolites, such as prostaglandins and Leukotrienes (LTs), drive neuroinflammation through Cyclooxygenase (COX) and Lipoxygenase (LOX) pathways, exacerbating ischemic injury. Conversely, the aim was to review the therapeutic potential of Specialized Pro-resolving Mediators (SPMs), including lipoxins, Resolvins (RVs), and protectins, that resolve inflammation and promote tissue repair. In addition, the roles of Peroxisome Proliferator-Activated Receptors (PPARs) and sphingolipid signaling in modulating oxidative stress, mitochondrial dysfunction, and neuronal survival were also addressed. Integrating recent advances in lipid biology and cerebral ischemia research, this review presents an overview of the role of lipid metabolism in disease progression and its potential as a target for new therapeutic interventions. These findings bridge the gap between basic science and clinical research, opening new doors for the treatment of cerebral ischemia.

## 1 INTRODUCTION

Stroke is a severe neurological condition with high prevalence, disability, and mortality, ranking second only to malignant neoplasms as a cause of death worldwide, with an estimated annual death toll of 6.5 million [1]. More than 3 million new strokes occur annually in China, of which almost 70% are ischemic strokes [2]. The pathophysiology of ischemic stroke is complex, involving such mechanisms as energy failure, excitotoxicity, oxidative stress, and inflammatory processes, leading to neuronal death and neurological deficits [3]. Lipids are a group of essential organic substances that perform several functions in the brain, including serving as substrates for energy, structural components of cell membranes, and cellular signal mediators [4]. Lipid metabolism is significantly altered following cerebral ischemia, encompassing synthesis, intermediate metabolism (modification, transport, and signaling), storage, and degradation [5]. Increasing evidence suggested that lipid metabolism plays a crucial role in cerebral ischemia and is closely linked to post-ischemic recovery [6]. Therefore, in this review, the alterations and impacts of lipid metabolism following cerebral ischemia are systematically reviewed, and possible therapeutic targets are identified with insights.

### 1.1 Overview of Cerebral Ischemia

Cerebral ischemia is a pathological condition induced by reduced or severed cerebral blood flow with resultant breakdown in the supply of oxygen and nutrients, hence causing damage to brain tissue. Concerning disease course and pathological features, cerebral ischemia can be classified as Transient Ischemic Attack (TIA), ischemic stroke, and chronic cerebral ischemia. Ischemic stroke secondary to acute arterial occlusion is characterized by focal neurological deficits, including hemiparesis, dysarthria, sensory loss, aphasia, and visual disturbances [7]. TIA was previously defined as neurological symptoms that resolve completely within 24 hours, but with the advancement in imaging techniques, even transient symptoms with Magnetic Resonance Imaging (MRI) evidence of restricted diffusion are being referred to as ischemic stroke [8]. Chronic cerebral ischemia, or prolonged impairment of cerebral perfusion, may lead to vascular cognitive impairment. Its pathogenesis is due to systemic, cardiac, or regional vascular diseases, but its precise pathophysiological mechanisms are not yet fully understood [9].

The initial treatment goal for acute ischemic stroke is the restoration of cerebral perfusion as early as possible, either by a procedure like intravenous thrombolysis, endovascular thrombectomy, or both [7]. In animal studies, the transient Middle Cerebral Artery Occlusion (tMCAO) model is commonly employed to simulate ischemia-reperfusion injury, achieved by inserting a filament into the external carotid artery to occlude the middle cerebral artery temporarily for blood flow [10]. Chronic cerebral ischemia models can be produced by Bilateral Common Carotid Artery Occlusion (BCCAO) or stenosis [9]. Additionally, *in vitro* experiments frequently employ the Oxygen-Glucose Deprivation (OGD) model to simulate the cellular pathophysiology of ischemic stroke [11].

### 1.2 Importance of Lipid Metabolism in the Brain

Lipids are a group of organic compounds insoluble in water but soluble in organic solvents [12]. Hydrophobic or amphipathic molecules, in themselves, most likely form their structures through the condensation of thioesters and/or isoprene units by carbonyl anions or carboxyl cations [13]. According to the International Lipid Classification and Nomenclature Committee, eight major lipid classes are fatty acyls, glycerolipids, glycerophospholipids, sphingolipids, saccharolipids, polyketides (which are products of ketoacyl subunit condensation), sterol lipids, and prenol lipids (which are products of isoprene subunit condensation) [14].

The brain's lipid content is primarily composed of phospholipids, cholesterol, sphingolipids, and glycerolipids [15]. Lipids are uniformly distributed in neurons, glial cells, and myelin, and they play both significant structural and functional roles. The most critical lipid pathways include several essential processes [16]. Fatty Acid (FAs) synthesis and β-oxidation are essential pathways involved in lipid synthesis as well as energy generation [17]. Cholesterol synthesis and regulation are necessary for neuronal cell membrane growth and repair [18]. The metabolism of sphingolipids and ceramide-mediated signaling directly influence neuronal function and communication [19]. Phospholipid remodeling and the production of eicosanoids are also vital components of brain lipid metabolism and involve maintenance of membrane stability and the transmission of signals [20].

### 1.3 Methods

A systematic search was conducted on PubMed and Web of Science until March 2025 to collect, select, and analyze literature related to cerebral ischemia and lipid metabolism. The search strategy was developed by integrating controlled vocabulary (MeSH terms) with natural language terms, informed by previously published reviews.

The search terms included:

((cerebral ischemia OR ischemic stroke OR brain ischemia) AND ((hyperacute phase AND (energy failure OR lipid peroxidation)) OR (subacute phase AND (inflammation OR lipid mediators)) OR (chronic phase AND (remodeling OR repair)) OR (pro-inflammatory pathways OR pro-inflammatory cytokines) OR (anti-inflammatory pathways OR pro-resolving pathways OR specialized pro-resolving mediators) OR (apoptosis OR autophagy) OR (lipid metabolism OR lipid profile OR lipidomics) AND (neurons OR neural stem cells OR neuroglia OR astrocytes OR microglia) OR (therapeutic targets AND (lipid metabolism OR lipid profile OR lipidomics)))).

## 2 DYSREGULATION OF LIPID METABOLISM IN CEREBRAL ISCHEMIA

**Fig. (1):**
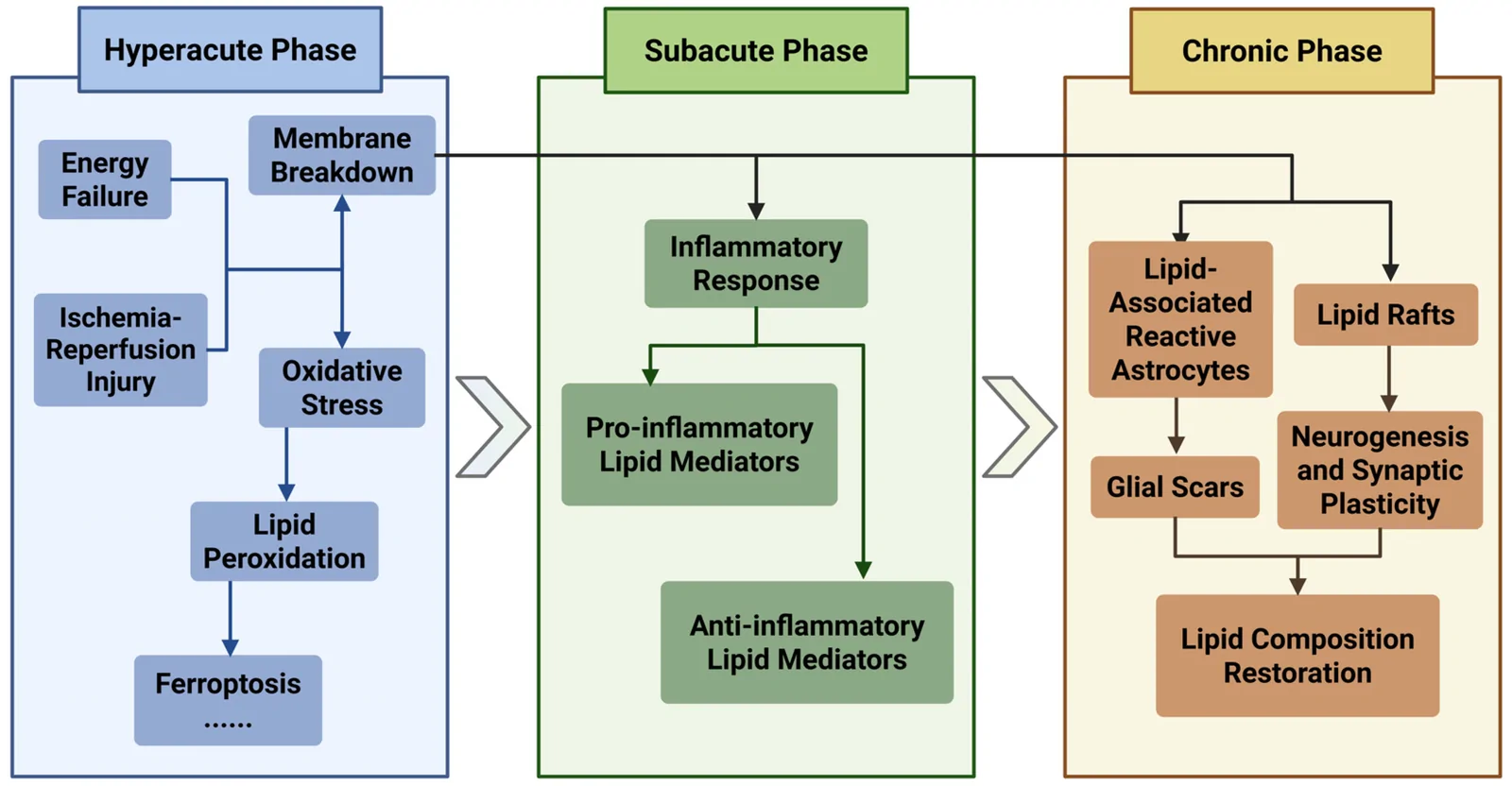
The pathological process of cerebral ischemia and lipid metabolism. Figure created in BioRender. Bai, Q. (2025). https://biorender.com/zc39ist.

 Following cerebral ischemia, extensive alterations in lipid metabolism occur, varying from the hyperacute to the subacute and chronic phases (Fig. **1**). These changes are closely correlated with disturbances in energy metabolism, the inflammatory response, and tissue repair [6, 21].

### 2.1 Hyperacute Phase: Energy Failure and Lipid Peroxidation

During the hyperacute phase of cerebral ischemia (seconds to minutes), the occlusion of the vasculature results in regional hypoxia-ischemia and energy deprivation [22]. Medium-chain FAs (*i.e*., octanoate) are an alternative source of energy and supply approximately 20% of the brain's energy through FA oxidation [23]. The breakdown and oxidation of medium-chain FAs can be regulated by the body *via* negative feedback mechanisms to reverse energy depletion and maintain homeostasis [24].

Long-term ischemia disrupts ion homeostasis in the ischemic core, leading to the release of glutamate on a large scale and calcium overload through N-Methyl-D-Aspartate receptors (NMDA). This further activates Phospholipase A2 (PLA2), Phospholipase C (PLC), proteases, and endonucleases [25-28]. The activation of PLC and PLA2 leads to degradation of neuronal membrane glycerophospholipids, for example, Polyphosphoinositides (PIPs), leading to rapid decreases in levels of Phosphatidylinositol 4,5-bisphosphate (PIP2), Phosphatidylinositol 4-phosphate (PIP), and Phosphatidylcholine (PC) with increased levels of Platelet-Activating Factor (PAF) and Lysophosphatidylcholine (LPC) [29-31]. For instance, in one study that used basilar artery electrocoagulation and BCCAO to induce global ischemia, it was found that the content of PIP2 and PIP significantly decreased, while LPC levels increased within 5 minutes [32]. Activation of phospholipase also increases Free Fatty Acids (FFAs), Diacylglycerol (DG), and Triacylglycerol (TG) with the fast release of AA [33, 34]. Shinichi Yoshida *et al.* have noted elevation of FFAs and DGs, including Diacylglycerol-Stearic Acid (DG-SA) and Diacylglycerol-Arachidonic Acid (DG-AA), to a significant degree within 0-15 minutes of ischemia in Wistar rats [35]. AA also stimulates Sphingomyelinase (SMase), which hydrolyzes Sphingomyelin (SM) to form Ceramide (Cer) [36]. Cerebral ischemia for 10 minutes transiently activates the SM-Cer pathway, leading to Cer accumulation in the hippocampus of Sprague-Dawley rats [37]. Cer induces apoptosis through inhibition of mitochondrial electron transport and cytochrome c release [38, 39].

Oxidative stress also plays a key role in the acute phase. The unregulated formation of Reactive Oxygen Species (ROS) drives lipid peroxidation, which compromises the integrity of organelles and cell membranes and speeds neuronal injury [40]. ROS levels are extraordinarily elevated in brain cells and tissues following Middle Cerebral Artery Occlusion (MCAO) and OGD [41]. Specifically, ROS attack polyunsaturated FAs with carbon-carbon double bonds, inducing chain reactions to form lipid peroxides and toxic aldehydes such as Malondialdehyde (MDA) and 4-Hydroxy-2-Nonenal (4-HNE), which compromise membrane structure and function [42, 43]. Lipid peroxidation is also a key mechanism of ferroptosis [44], where lipid peroxides (such as phospholipid hydroperoxides) interact with iron ions to form lipid radicals, further exacerbating the lipid peroxidation reaction, ultimately leading to cell membrane damage and cell death [45]. Excessive Reactive Oxygen Species (ROS) and Reactive Nitrogen Species (RNS) can disrupt mitochondrial membrane permeability, impair the function of the electron transport chain, leading to electron leakage, reduced ATP production, and release of cytochrome c, which exacerbates neuronal damage [40, 46]. Moreover, excessive ROS and RNS can directly trigger the secretion of inflammatory molecules and the activation of inflammasomes [47]. ROS can activate various inflammatory signaling pathways, such as the Nuclear Factor-κB (NF-κB) pathway. Once activated, NF-κB translocates to the nucleus, promoting the transcription and expression of inflammatory factors such as Tumor Necrosis Factor-α (TNF-α) and Interleukin-1β (IL-1β) [48]. RNS is involved in the caveolin-1 and Matrix Metalloproteinase (MMP) signaling pathways, which contribute to the progression of neuroinflammation and disruption of the Blood-Brain Barrier (BBB) [47, 49]. It has also been reported that ROS can induce DNA damage and impair DNA repair, leading to genomic instability and exacerbating inflammatory responses [50]. The crosstalk between DNA damage and inflammation, in turn, leads to ROS accumulation [51], and this vicious cycle further exacerbates the damage following cerebral ischemia.

### 2.2 Subacute Phase: Inflammation and Lipid Mediators

During the subacute phase (minutes to hours), inflammation predominates due to BBB disruption and activation of various cell types [52]. Repeated activation of phospholipase leads to massive release of AA, which is metabolized *via* the COX and LOX pathways to yield a host of lipid mediators [53-55].

#### 2.2.1 Pro-inflammatory Lipid Mediators

After cerebral ischemia occurs, prostaglandins (such as Prostaglandin E2 (PGE2), Prostaglandin D2 (PGD2)), LTs, and thromboxanes (such as Thromboxane B2 (TXB2)) are gradually produced. These mediators further exacerbate brain damage by promoting vascular permeability, recruiting immune cells, and enhancing inflammatory responses [55]. For example, in brain ischemia models such as Unilateral Common Carotid Artery Occlusion (UCCAO), MCAO, and BCCAO, the levels of PGE2 and PGD2 in the brain tissue on the side of injury are significantly increased [56-58], which promotes the infiltration of inflammatory cells and causes edema [59, 60]. The levels of LTs are also further increased, triggering neuroinflammation [61, 62].

#### 2.2.2 Anti-inflammatory Lipid Mediators

Subsequently, anti-inflammatory lipid mediators such as RVs, protectins, and lipoxins are generated in the late phase of inflammation. These mediators inhibit inflammatory cell infiltration and promote resolution, exerting protective effects [63]. For example, in both tMCAO and permanent Middle Cerebral Artery Occlusion (pMCAO) models, administration of Lipoxin A4 (LXA4) analogs or Neuroprotectin D1 (NPD1) significantly ameliorates cerebral edema and BBB disruption following ischemia. It reduces the infarct area [64-66].

#### 2.2.3 Sphingolipid Metabolism

Sphingolipid metabolism undergoes significant changes during the subacute phase. Cer levels rise, while its downstream metabolite Sphingosine-1-Phosphate (S1P) transiently increases, maintaining a "sphingolipid rheostat" balance that regulates cell survival and death [67, 68]. S1P receptor agonists reduce infarct volume and improve neuronal survival in tMCAO models [69]. S1P, a signaling lipid that enhances growth and survival in various cell types [70], mediates glial and inflammatory responses, maintains endothelial function, and drives post-ischemic neurogenesis and recovery through sphingosine-1-phosphate receptors (S1PR1, S1PR2, S1PR3, *etc*.) [71-73].

### 2.3 Chronic Phase: Remodeling and Repair

In the chronic phase (days to weeks), lipid metabolism changes are primarily associated with tissue repair and functional recovery [74]. Edema resolution, reperfusion, neurogenesis, and synaptic plasticity are key aspects of this phase [75-77].

#### 2.3.1 Changes in Lipid Composition

Within 1-3 days of reperfusion, PC and Phosphatidyl Ethanolamine (PE) levels gradually recover, while LPC and Cer levels decline [30]. The majority of lipid concentrations, however, return to baseline by 14-28 days following ischaemia [30].

#### 2.3.2 Role of Lipids in Gliosis and Neurogenesis

Lipids regulate glial cell function and the differentiation of Neural Stem Cells (NSCs), and are involved in glial scar formation and neurogenesis. In cerebral ischemia, lipids released from injured neurons are engulfed by astrocytes and become Lipid-Associated Reactive Astrocytes (LARAs) [78, 79]. Astrocytes are involved in the formation of glial scars, which prevent over-inflammation and progression of injury [80]. The recovery of Phosphatidylethanolamine (PE) and Phosphatidylserine (PS) is linked with neuronal regeneration. PE interacts with ubiquitin-like proteins such as ATG8 in autophagy, a mechanism crucial for the formation of autophagosomes [81, 82]. Autophagy degrades dysfunctional organelles, axons, and myelin, protecting neurons and inhibiting oxidative damage and inflammation [83]. Exposure of PS is known to induce post-injury fusion [84], where lipid peroxidation regulates the externalization of PS to aid in axonal fusion and repair [85].

#### 2.3.3 Role of Lipid Rafts in Synaptic Plasticity and Recovery

Lipid rafts, membrane microdomains enriched in cholesterol and sphingolipids, play a crucial role in synaptic plasticity and signaling [86-88]. Functional recovery is directly correlated with post-ischemic reorganization of lipid rafts [89, 90]. Signaling molecules such as glycoprotein M6a, ephrin B receptors, and netrin-1 receptors accumulate in lipid rafts, influencing actin networks and providing a microenvironment for new synapse formation [91-93]. Cholesterol in lipid rafts is required for synaptogenesis, and loss of it can lead to synapse loss [88, 94].

The dynamic interplay between cerebral ischemia progression and lipid metabolic alterations across three phases: hyperacute, subacute, and chronic. In the hyperacute phase (seconds to minutes), energy failure, lipid peroxidation, and phospholipase activation lead to membrane phospholipid breakdown, release of AA, and ceramide accumulation, exacerbating oxidative stress and mitochondrial dysfunction. During the subacute phase (minutes to hours), AA metabolites drive neuroinflammation, while SPMs and S1P promote resolution. In the chronic phase (days to weeks), lipid composition remodeling, autophagy, and lipid raft reorganization support synaptic plasticity, gliosis, and neurogenesis.

## 3 LIPID-MEDIATED SIGNALING PATHWAYS IN CEREBRAL ISCHEMIA

### 3.1 Pro-inflammatory Pathways

#### 3.1.1 Role of AA Metabolites in Neuroinflammation

When cerebral ischemia occurs, the level of AA increases sharply, and its metabolites play a significant role in the neuroinflammatory process. Some experiments have revealed that the levels of AA and its derivatives are typically higher in ischemic areas of the brain following cerebral ischemia, a process confirmed in numerous animal models [57, 61, 95].

AA is an unsaturated FA. After cerebral ischemia occurs, the release of glutamate and calcium overload mediated by NMDA receptors leads to the activation of a series of enzyme systems, including PLA2 and PLC, which release free AA from phospholipids [96, 97]. Subsequently, AA is metabolized into PGE2, PGD2, LTs, thromboxane A2 (TXA2), and other pro-inflammatory substances through the COX or LOX pathways [55]. Specifically, PGE2 can promote the activation of inflammatory cells, tissue edema, and the release of inflammatory factors through its receptors EP1, EP2, EP3, and EP4 [98-100]. PGD2 affects vascular permeability and the chemotaxis of inflammatory cells through its receptors DP1 and DP2 [101, 102]. LTs enhance the inflammatory response and the recruitment of immune cells through their receptors CysL, promoting vascular permeability and leukocyte-driven processes [103]. Additionally, Cys-LT receptors can trigger the release of inflammatory substances, resulting in neuronal injury and neurodegeneration [104]. TXA2 promotes platelet aggregation and vascular contraction through its receptor TP, and is involved in oxidative stress and inflammatory processes in cerebral ischemia [105].

#### 3.1.2 Activation of NF-κB and Cyclooxygenase-2 (COX-2)

During cerebral ischemia, NF-κB and COX-2 are activated, resulting in the excessive production of inflammatory mediators such as prostaglandins [60, 106]. COX exists in two major isoforms, COX-1 and COX-2 [107]. The COX-1 isoform is constitutively expressed in most mammalian cells and tissues to maintain normal homeostasis, while COX-2 is an inducible enzyme and the predominant isoform expressed in neurons, typically expressed at low levels under normal physiological conditions [108]. Following cerebral ischemia, the activation of NMDA receptors can lead to the expression of COX-2 in neurons and the synthesis of several prostaglandins [53, 54]. Studies have shown that the generation of these prostaglandins has a critical function in neuroinflammation and disease processes following cerebral ischemia [60].

The role of AA and its derivatives with cerebral ischemia is complex, wherein COX-1 and COX-2 play different roles in modulating inflammation. COX-1 is theorized to be involved in the brain's housekeeping functions, regulating blood flow and vasodilation [109] and possibly playing a beneficial regulatory role against cerebral ischemia. COX-2 plays largely pathological roles. The reaction of free radical nitric oxide (NO) with the COX-2 produced under cerebral ischemia can augment the generation of toxic prostaglandins and ROS, thereby aggravating neuronal injury [110]. Inhibition of AA and its derivatives would be a highly valuable approach against cerebral ischemia. COX-2 plays a key part in ischemic brain injury in animal models [60], and selective pharmacologic inhibition of COX-2 action has been an interesting therapeutic target for stroke. Potent and highly selective inhibitors of COX-2 can inhibit prostaglandin production [111] and reduce focal cerebral ischemic insult in rats. However, selective COX-2 inhibitors have many side effects and render targeting of essential pro-inflammatory prostaglandin receptors more specific [112].

### 3.2 Anti-inflammatory and Pro-resolving Pathways

#### 3.2.1 SPMs and Their Role in Inflammation Resolution

SPMs are a unique class of lipid mediators, including lipoxins, RVs, protectins, and Maresins (MaR) [113, 114]. SPMs play a significant role in anti-inflammatory and pro-resolving activity during the inflammatory process after cerebral ischemia [115]. Various experiments have demonstrated that in MCAO, BCCAO, and OGD *in vitro*, SPM administration is able to modulate the inflammatory response, promote the resolution of inflammation, and support tissue repair [116-119]. SPMs can inhibit the release of inflammatory mediators, reduce cellular damage, and promote repair and regeneration of damaged tissues [120]. Also, SPMs correct the inflammation response, inhibit excessive release of inflammatory mediators, and block the persistence of the inflammatory response, thereby protecting neurons from continued damage [113].

Action mechanisms of SPMs involve the regulation of multiple signaling pathways and cytokines. SPMs can suppress the activation of central protein kinases in the inflammatory cascade, such as NF-κB, thereby inhibiting the synthesis and release of inflammatory mediators [121]. SPMs exert anti-inflammatory effects through the ALXR-mediated pathway, significantly inhibiting the release of pro-inflammatory cytokines and inflammatory mediators [116]. SPMs can activate the Kelch-like ECH-associated protein 1-nuclear factor erythroid 2-related factor 2 (Keap1-Nrf2) pathway [122]. With the help of Keap1, the dissociation of the complex allows Nrf2 to translocate to the nucleus [123, 124], where it can bind to the promoters of specific antioxidant genes such as HO-1, ARE, and GPx, maintaining redox balance and inhibiting the expression of pro-inflammatory cytokines (such as TNF-α, IL-6), thereby exerting anti-inflammatory and antioxidant stress effects [125]. In addition, SPMs can regulate apoptosis and autophagy pathways, promoting the repair and clearance of damaged organelles, thereby helping to restore normal cellular function [126]. Specifically, lipoxins can inhibit the translocation of 5-lipoxygenase and the biosynthesis of LTs by suppressing pathways such as NF-κB and MMP-9; they can also inhibit neutrophil infiltration and lipid peroxidation levels; and they can inhibit the activation of microglia, reducing pro-inflammatory cytokines while increasing anti-inflammatory cytokines [64, 116, 127, 128]. RVs can reduce leukocyte infiltration and prevent the expression of pro-inflammatory genes by activating Akt and inhibiting NF-κB, and they can also promote the clearance of apoptotic cells, exerting anti-inflammatory and neuroprotective effects [129, 130]. Moreover, protectins can downregulate pathways such as TLR4/ NF-κB, thereby inhibiting the infiltration of polymorphonuclear leukocytes, the activation of the complement system, and the expression of inflammatory factors [131, 132].

SPMs play an important role during the process of resolving inflammation. In addition to preventing the persistence of inflammation, SPMs promote the resolution process of inflammation and tissue repair, thereby protecting harmed tissues and preventing further tissue damage caused by inflammation [133]. Therefore, lipoxins, RVs, protectins, and MaR might be an effective approach against neuroinflammation following cerebral ischemia.

#### 3.2.2 Activation of PPARs

PPARs are a subclass of nuclear receptor transcription factors that are essential in regulating inflammatory responses and metabolic pathways [134]. Activation of PPAR has the potential to reverse post-ischemic inflammation and trigger tissue repair [135]. Experiments have shown that in animal models of cerebral ischemia, dual agonists of PPARα and PPARγ treatment can significantly suppress inflammatory factors and vascular inflammation, triggering post-ischemic recovery [136, 137]. PPARs regulate gene expression, which affects glucose uptake, lipid metabolism, and inflammation [138]. Overall, PPAR activation favors balance within the inflammatory process by promoting mitochondrial function, inhibiting microglial activation, reducing the release of inflammatory mediators, and protecting neurons from being damaged as a result of inflammation [139].

Activation routes of PPARs include the regulation of numerous pathways of signaling and gene expression [140]. Activation of PPAR suppresses inflammatory gene transcription, inhibits NF-κB- and AP-1-induced neuroinflammation, and reduces the synthesis and secretion of cytokines, chemokines, and adhesion molecules, thereby attenuating the inflammatory response [141-143]. PPAR activation also prevents oxidative stress by pathways involving the MAPK and AMPK signaling cascades [144]. For example, Docosahexaenoic Acid (DHA) can activate PPARγ to inhibit ERK and activate the AKT signaling pathway to regulate microglial polarization and inhibit neuroinflammation [145]. In addition, PPAR activation is involved in regulating apoptosis and autophagy processes and promotes healing and clearance of damaged organelles, contributing to the well-being of cells [146].

Following cerebral ischemia, PPAR activation not only inhibits the sustainability of the inflammatory response but also aids in the resolution of inflammation and tissue repair. They play a role in regulating lipid metabolism, anti-inflammatory processes, and pro-resolving pathways and therefore present novel therapeutic potential for the regulation of inflammatory responses and tissue repair.

### 3.3 Apoptosis and Autophagy Pathways

#### 3.3.1 Ceramide-Mediated Apoptosis and Mitochondrial Dysfunction

Cerebral ischemia induces significant changes in Cer levels, particularly during ischemia-reperfusion. For example, after MCAO and reperfusion, the time course of appearance of phospholipids in the hippocampal region changes, and Cer signaling in the CA1 region of rats is significantly enhanced [147]. Another study on gerbil cerebral ischemia determined that after BCCAO for 5 minutes, inducing lethal cerebral ischemia, the content of SM in the hippocampus fell dramatically within a short period, with the formation of Cer, and this change was irreversible [148]. Cer is a bioactive sphingolipid with a strong correlation to apoptosis [149]. The rise of Cer after ischemia is largely due to endogenous long-chain Cers, such as Cer (d18:1/16:0) and Cer (d18:1/18:0) [67]. Such an increase may have the potential to exacerbate cellular damage, particularly in astrocytes [37]. The rise of Cer may also be responsible for mitochondrial dysfunction by disrupting intracellular calcium homeostasis and inducing oxidative stress, which can result in apoptosis [150].

Therapeutic strategies for Cer accumulation may include inhibition of superoxide generation, modulation of inflammation, and reduction of lipid peroxidation [151, 152]. Inhibition of Jun N-terminal Kinase (JNK) and Protein Phosphatase 2A (PP2A) may reduce cell death [37]. In addition, the inhibition of SMase and serine palmitoyltransferase can regulate Ceramide synthesis and lower Ceramide levels [153]. In the case of mitochondrial dysfunction from Cer accumulation, target points may be SIRT3 and other analogous molecules to maintain mitochondrial function and reduce cellular damage [154].

#### 3.3.2 Role of Lipids in Autophagy and Clearance of Damaged Organelles

In cerebral ischemia, various bioenergetic and functional cellular alterations play a crucial role in processes of cell survival and death. A precipitous decline in ATP is one of the most critical factors, leading to the disruption of ion gradients and membrane depolarization, with the activation of phospholipases and the start of phospholipid degradation and accumulation of FFAs [22, 34]. It is also involved in intracellular disruption of calcium homeostasis, leading to a cascade of intracellular events, including excessive release of glutamate, activation of proteases and lipases, and inhibition of protein synthesis [155]. Endoplasmic reticulum stress and mitochondrial injury are major pathogenic mechanisms [156-158]. Overloading of mitochondria with calcium leads to dysfunction by increasing membrane permeability and the activation of the release of cytochrome c, and activation of the process of apoptosis [159]. Furthermore, the deposition of FFAs and alteration of membrane phospholipid composition can also be harmful to the inner mitochondrial membrane, leading to mitochondrial dysfunction, including mitochondrial uncoupling, inhibition of respiratory chain enzymes, and reduced oxidative phosphorylation [160]. Studies using carotid artery occlusion models have shown that in the early stages of ischemia, the activation of PLA2 leads to a decrease in the content of PC, PE, and cardiolipin in mitochondria. These changes are accompanied by a reduction in mitochondrial respiratory state and inhibition of COX and ATP synthase, processes that depend on cardiolipin content and FAs composition [161, 162].

Among the therapies proposed, some of them include protection of mitochondria from damage through statins, individual long-chain ω-3 FAs, and triheptanoin [163-165]. The drugs and agents were believed to protect mitochondria from damage. Endogenous cannabinoid receptor agonists and hydrogen have also been shown to have potential in protecting neurons and mitochondrial function [166, 167]. In addition to the mentioned pathway, blocking the lysophosphatidic acid-phosphatidic acid autotaxin pathway or forcing myelination *via* exogenous healthy mitochondria are also potential treatments for mitochondrial injury [168], which represent good avenues for the reversal of cellular injury caused by cerebral ischemia.

## 4 CHANGES IN LIPID METABOLISM IN DIFFERENT CELL TYPES FOLLOWING CEREBRAL ISCHEMIA

### 4.1 Neurons

**Fig. (2):**
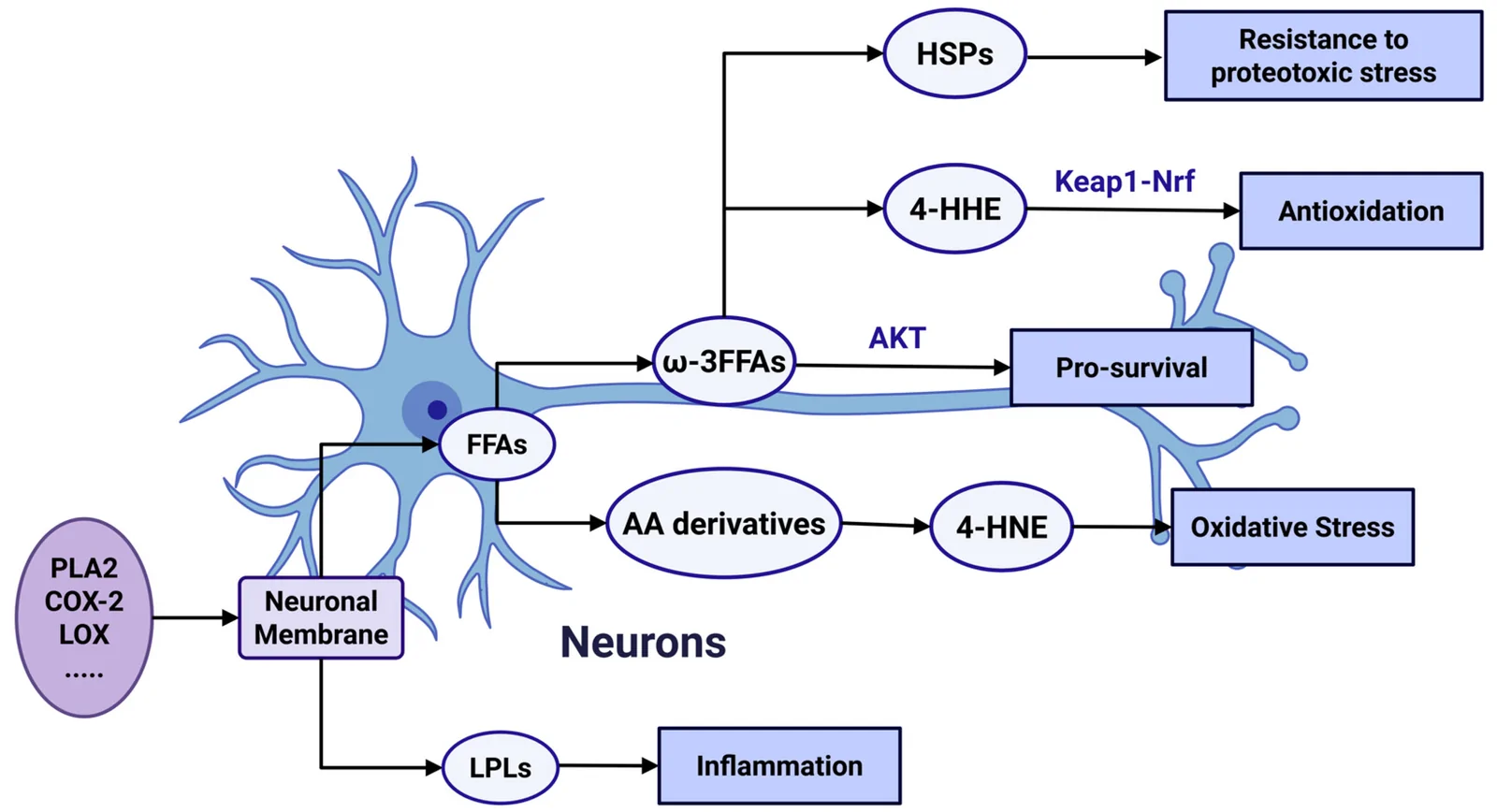
The role of neuronal membranes in cerebral ischemia. Figure created in BioRender. Bai, Q. (2025). https://biorender.com/9ups3vc.

The neuronal membrane is the primary mediator of stress responses induced by ischemia (Fig. **2**). Activation of phospholipases, particularly PLA2, triggered by ischemia, leads to the hydrolysis of phospholipid backbones, releasing FFAs and Lysophospholipids (LPLs) [169]. LPLs are direct precursors of PAF, a potent inflammatory mediator that exerts its effects by binding to the PAF receptor [170]. The oxidative compounds generated by these pathways, especially derivatives of AA and DHA, affect neuronal survival following cerebral ischemia. Derivatives of AA mainly promote neuronal death after cerebral ischemia, so inhibiting COX can achieve neuroprotective effects [111]. Among the metabolites produced by non-enzymatic oxidation of AA, 4-HNE is a significant biomarker of oxidative stress. Changes in its concentration influence cell survival and apoptosis. For instance, low 4-HNE levels (<2 μM) stimulate cell growth and survival, but higher levels (10-60 μM) are genotoxic, causing sister chromatid exchange, micronuclei, and DNA fragmentation and inhibiting key enzymes involved in physiological processes [171].

Moreover, the release and metabolism of FFAs play a significant role in cell survival and death following cerebral ischemia (Fig. **2**). Oxygenases, including COX-2 and LOX, catalyze the production of RVs, protectin D1 (PD1), and MaR from ω-3 FFAs such as DHA and Eicosapentaenoic Acid (EPA) [172]. In the presence of aspirin, COX-2 targets DHA to generate aspirin-induced RVs, which possess strong anti-inflammatory and immunomodulatory activities, regulate leukocyte migration, and suppress cytokine expression in cultured glial cells [16]. DHA also modulates the AKT signaling pathway positively and induces pro-survival pathways [173]. Its beneficial effects in ischemia include the action of Uncoupling Proteins (UCPs), mitochondrial transporters that maintain proton conductance across the inner mitochondrial membrane. Increased expression and activity of UCP2 are associated with neuronal survival post-stroke [174]. In addition, non-enzymatic oxidation of DHA produces 4-Hydroxyhexenal (4-HHE), which in turn activates the Keap1-Nrf pathway to increase antioxidant protein synthetic genes and activate an adaptive response to oxidative/ electrophilic stress [175]. Electrophilic lipids could also enhance cellular capacity to manage proteotoxic stress by inducing intracellular Heat Shock Proteins (HSPs) [176].

 The neuronal membrane dynamics during ischemia. Hypoxia-ischemia triggers PLA2 activation, leading to phospholipid hydrolysis, release of FFAs, and generation of LPLs and lipid peroxidation products (*e.g*., 4-HNE). Pro-inflammatory AA metabolites (*via* COX/LOX pathways) exacerbate neuroinflammation and oxidative stress, while ω-3 fatty acid-derived RVs and protectins counteract these effects. Mitochondrial dysfunction, calcium overload, and the production of ROS/RNS further disrupt membrane integrity. The figure also highlights lipid raft-mediated synaptic signaling and the dual roles of lipid mediators in regulating neuronal survival and apoptosis.

### 4.2 Astrocytes

Astrocytes are the most prevalent glial cells in the Central Nervous System (CNS). Astrocytes, from an inflammatory perspective, undergo tremendous structural and functional alterations following ischemic stroke [177] and have dual functions in CNS disease, either facilitating or preventing CNS recovery [178-180]. For example, during the subacute phase of ischemic brain injury, *de novo* lipogenesis in astrocytes increases IL-33 production around the ischemic area, promoting BBB repair and improving long-term functional recovery [181]. During OGD conditions, the enhanced expression of Cytochrome P450 4A (CYP4A) in astrocytes enhances 20-Hydroxyeicosatetraenoic acid (20-HETE) production, promoting endothelial cell proliferation, tube formation, and migration and contributing to neurovascular remodeling and recovery of function following ischemic stroke [182]. Reactive astrogliosis in the peri-infarct region also acts to limit the spread of the injury region, remodel tissue, and modulate local immune responses [183]. However, reactive astrogliosis turns into glial scar formation during the chronic phase (1-4 weeks post-stroke), which prevents neural regeneration and functional recovery [184].

From the energy metabolism perspective, neurons and astrocytes are a unified system in brain energy metabolism. FA's metabolism is a significant area of neuron-astrocyte interaction. Neurons are not capable of forming Lipid Droplets (LDs) in general and have limited capacity to oxidize FAs for energy generation in mitochondria [185]. Astrocytes, however, can form LDs and produce antioxidants, thereby managing oxidative stress [186]. Specifically, during cerebral ischemia, hyperactive neurons release toxic lipids in ApoE-positive lipid particles, which are taken up by neighboring astrocytes *via* endocytosis. These transferred FAs serve as metabolic intermediates, enhancing mitochondrial oxidation in astrocytes and detoxifying neurons [78]. However, the lipid acceptance capacity of astrocytes is limited. Long-Chain Acylcarnitines (LCACs), showing notable changes in the ischemic penumbra and peripheral blood of tMCAO mice, exacerbate mitochondrial damage in astrocytes, reduce energy supply to neurons, and enhance neuronal damage [187].

Glycerol Triacetate (GTA) administration promotes BBB repair and functional recovery after ischemic stroke by enhancing astrocytic lipogenesis and IL-33 expression [188]. Exogenous gangliosides are protective against neuroinflammation and gliosis [189]. Deuterated linoleic acid polyunsaturated fatty acids (D4-Lnn) reduce hypoxia-induced elevation of cytosolic calcium, inhibit excessive production of ROS, enhance protective gene expression, and inhibit damage-producing protein production [190]. Valproic Acid (VPA) provides neuroprotection during the recovery from ischemic stroke through the inhibition of Histone Deacetylases (HDACs) and the activation of Hsp70.1B, as well as the prevention of glial scar formation [191]. D4-Lnn activates astrocytic phosphatidylinositol-calcium signaling pathway, inhibits OGD-induced elevation of calcium, reduces excessive ROS generation, enhances protective gene expression, and inhibits production of deleterious proteins [192].

### 4.3 Microglia

Microglia are resident immune cells of the CNS and play an important role in brain homeostasis [193]. Microglia become activated and transform into highly metabolically and phenotypically altered cells under pathological situations such as stroke. During the early stages of cerebral ischemia, microglia are anti-inflammatory, whereas with the passage of time, they acquire a pro-inflammatory phenotype [194]. This transformation is facilitated by metabolic adaptations to meet the high energy demands of morphological remodeling, phagocytosis, and cell division [195, 196]. In ischemic stroke models, microglia adapt to energy demands by enhancing glycolysis, lactate production, and the pentose phosphate pathway, while sacrificing mitochondrial oxidative phosphorylation. These metabolic changes promote lipid synthesis, supporting synthetic processes such as cell proliferation [197-199].

Single-cell transcriptomics has identified a Stroke-Associated Myeloid Cell (SAMC) population that is similar to the well-studied Disease-Associated Microglia (DAM) subset [200]. SAMCs exhibit a phagocytic phenotype, upregulate genes related to lipid metabolism, and display a gene expression profile indicative of high lysosomal activity [201]. Experimental evidence suggests that microglial LDs often contact lysosomes, indicating lipophagy [197]. Nevertheless, some of the microglia may accumulate LDs from defective lipophagy or low cytoplasmic lipase activity and become defective foam cells [202]. This may be related to an incompetent or defective autophagic flux leading to injury during phagocytosis [202]. Long-term intracellular lipid deposition may signal cellular exhaustion and injury [196].

During hypoxia or stroke, microglia were found to accumulate more LDs and exhibit expression of fatty acid-binding protein 2 (PLIN2). Lipid Droplet-associated Microglia (LDRMs) also express a pro-inflammatory phenotype with differentially regulated lipid metabolism genes that alter the content of cholesterol esters and TG and, in turn, enhance inflammation [203]. TREM2 is highly expressed in activated microglia post-stroke, and its deficiency impairs phagocytosis and promotes cholesterol ester accumulation, LDs formation, and PLIN2 upregulation. TREM2 knockdown also increases lipid synthesis while reducing cholesterol clearance and lipid hydrolysis, affecting microglial phenotype. Additionally, downregulation of the TGF-β1/Smad2/3 signaling pathway drives microglia toward a pro-inflammatory phenotype [204]. S1P also increases microglial phagocytosis against TREM2 [205], but higher levels of TGF-β1 inhibit the formation of LDs and produce their neuroprotective effects [206]. Furthermore, food ω-3 PUFAs (Polyunsaturated Fatty Acids) are also anti-ischemic stroke drugs because they inhibit microglia-induced neuronal damage and inflammation [207].

### 4.4 Neural Stem Cells

NSCs are major contributors to endogenous regenerative responses to cerebral ischemia. Ischemia activates NSCs and precursor cells in the adult brain to cause neurogenesis and oligodendrogenesis, thereby holding a promise for ischemic brain repair [208]. Lipid metabolism plays a critical role in the process, and its disturbance can trigger oxidative stress and inflammation, exacerbating neural injury. Additionally, it regulates neural regeneration through multiple pathways.

Post-cerebral ischemia, oxidative stress, and inflammation also activate Apoptosis Signal-regulating Kinase 1 (ASK1), which promotes the differentiation of endogenous NSCs into neurons [209]. Microglia also release the chemokine CXCL13, which acts on the CXCR5 receptor to facilitate the migration of neuroblasts in neonatal mouse Subventricular Zone (SVZ) specimens, indicating that inflammation is a potent inducer of post-stroke neurogenesis [210]. In inflammatory diseases, NSCs and precursor cells in the SVZ secrete the endocannabinoid anandamide, which regulates glutamatergic signaling *via* the type 1 Cannabinoid Receptor (CB1R) and prevents excitotoxic injury [211]. Short-Chain Fatty Acids (SCFAs) regulate astrocyte activation *via* the SGK1/IL-6 signaling pathway, alleviating the depletion of Oligodendrocyte Precursor Cells (OPCs) [212]. PGE1 treatment significantly promotes the proliferation and migration of endogenous NSCs [213]. Moreover, overexpression of the *C. elegans* fat-1 gene that desaturates ω-6 PUFAs to ω-3 PUFAs promotes NSC proliferation and differentiation, migration of neuroblasts to ischemic regions, and their maturation into neurons as well as oligodendrogenesis [214].

## 5 THERAPEUTIC TARGETS OF LIPID METABOLISM IN CEREBRAL ISCHEMIA

In the context of cerebral ischemia, various medications have been developed to target multiple biochemical pathways, and it is essential to understand how different drugs affect these processes. For example, antioxidant strategies have been proposed using compounds such as edaravone and coenzyme Q10, which are purported to block lipid peroxidation [215, 216] and potentially interfere with ROS-generating enzymes to reduce oxidative stress [217]. There are also ways in which lipid mediators are affected by drugs, some inhibiting pro-inflammatory pathways related to lipids [111] while others enhance certain anti-inflammatory processes and those that are pro-resolving [115, 135]. Cholesterol-lowering therapies achieve neuroprotection through the pleiotropic effects of statins and by targeting enzymes involved in cholesterol metabolism [218]. Sphingolipid-targeted therapies improve stroke outcomes by modulating Cer and S1P signaling, as well as by using fingolimod [71]. In addition, combination treatments that include lipid modulators alongside reperfusion treatments are thought to have synergistic effects, offering a more comprehensive therapeutic strategy for cerebral ischemia (Table **1**).

## 6 FUTURE RESEARCH DIRECTIONS

Lipid metabolism plays a crucial role in the onset, progression, and recovery from cerebral ischemia, participating in cellular signaling, energy storage, and oxidative stress response, as well as controlling disease progression by modulating inflammation and neural repair processes [6]. The direction of future research should focus on the following aspects: Firstly, employing systems biology and lipidomics to map lipid metabolism in cerebral ischemia, identifying new biomarkers and therapeutic targets. Secondly, investigating the specific roles of neurons, astrocytes, microglia, and endothelial cells in lipid metabolism as well as how lipid dysregulation affects the neurovascular unit. Thirdly, overcoming the limitation of the BBB for lipid-targeted therapy and developing lipid-based nanotherapies for drug targeting. Personalized medicine should also consider the roles of genetic and epigenetic factors in lipid metabolism and stroke outcomes, tailoring lipid-modulating therapies based on individual metabolic profiles.

During the pathological process of cerebral ischemia, lipid metabolism-related enzymes undergo functional changes, such as the activation of phospholipases, leading to cell membrane phospholipid hydrolysis and affecting membrane stability [27]. Lipid peroxidation and oxidative stress can exacerbate neural damage during the early ischemic and reperfusion phases, and lipid metabolism abnormalities cause inflammatory responses, releasing mediators like prostaglandins and LTs, to further damage neural cells [55]. However, during the recovery phase, lipid metabolism supports repair by modulating inflammatory balance and by promoting neuronal regeneration [63]. Certain lipid metabolites exhibit neuroprotective effects through intracellular signaling pathways, which promote neuronal survival.

With the improved insight into lipid metabolic mechanisms, its application in the treatment of cerebral ischemia has broad prospects. Intervention in lipid metabolic pathways or specific lipid enzymes may result in the development of new therapeutic schemes. Nutritional therapy, such as adjusting the dietary ratio between ω-3 and ω-6 FAs, is also considered a possible measure for decreasing metabolic disturbance and exerting neuroprotection [229]. In the future, research will explore the role of lipid metabolism in cerebral ischemia from various aspects, confirming efficacy and safety by clinical evidence and providing a scientific foundation for individualized treatment.

## CONCLUSION

In summary, lipid metabolism is implicated in the complex and critical process of cerebral ischemia development and recovery. Lipid metabolism is involved in maintaining cell membrane stability, providing energy, and responding to oxidative stress, as well as modulating disease pathology and prognosis by affecting inflammation and neural repair processes. Following cerebral ischemia, dysregulation of lipid metabolism can both promote neural injury and offer new potential therapeutic targets for neuroprotection and repair. Future research can better explain dynamic changes in lipid metabolic networks, examine cell-type-specific lipid metabolic mechanisms, and develop targeted therapies, such as antioxidants, lipid mediator modulators, statins for cholesterol lowering, and sphingolipid-targeted therapies. Furthermore, integration of systems biology, lipidomics, and personalized medicine platforms can uncover new biomarkers and therapeutic targets to provide a scientific basis for the early diagnosis and individualized treatment of cerebral ischemia. The limitations of the current study lie in the fact that the dynamic changes in lipid metabolism during cerebral ischemia still require further clinical data support, and certain mechanisms (*e.g*., lipid raft reorganization) remain incompletely elucidated. Future studies should validate potential therapeutic targets using multi-omics technologies. Through multidisciplinary cooperation and clinical translational research, the modulation of lipid metabolism is poised to become a leading trend in cerebral ischemia treatment, opening new avenues for improved patient outcomes.

## Figures and Tables

**Table 1:** Therapeutic strategies and mechanisms in cerebral ischemia.

**Treatment Strategy**	**Therapeutic Drug**	**Treatment Target**	**Therapeutic** **Mechanism**
Anti-inflammatory	Non-steroidal anti-inflammatory drugs (NSAIDs)	Inhibit COX-2	Inhibition of COX enzymes to reduce prostaglandin synthesis [111].
Anti-inflammatory	SC51089, ONO-AE3-240	Inhibit EP1, EP3	Inhibition of PGE2 effects [219-221].
Anti-inflammatory	Lipoxin (SPMs)	Inhibit 5-lipoxygenase translocation, NF-κB, MMP-9	Suppression of neutrophil infiltration and lipid peroxidation levels; inhibition of microglial activation [64, 116, 127, 128].
Anti-inflammatory	Protectins (SPMs)	Activate Akt, inhibit NF-κB	Reduction of leukocyte infiltration; promotion of apoptotic cell clearance [129, 130].
Anti-inflammatory	Resolvins (SPMs)	Inhibit TLR4/NF-κB	Attenuation of polymorphonuclear leukocyte infiltration, complement system activation, and inflammatory cytokine expression [131, 132].
Anti-inflammatory	Fingolimod (S1P receptor modulator)	Antagonize S1P receptors	Modulation of S1P signaling to reduce circulating lymphocytes [71].
Anti-inflammatory	MHY908, Fenofibrate, GW7647, Pioglitazone, Rosiglitazone, INT131	Activate PPARs	Downregulation of the synthesis and release of cytokines, chemokines, and adhesion molecules [140-143].
Anti-inflammatory	Cottonseed oil (CSO)	Inhibit TLR4/NF-κB pathway	Reduction of microglial and astrocytic activation and inflammatory responses [222].
Antioxidant Stress		Activates the Nrf2 signaling pathway	Activation of the endogenous antioxidant stress response pathway, Nrf2 signaling, to alleviate oxidative stress damage caused by ischemic stroke [222].
Antioxidant Stress	β-hydroxybutyrate (BHB) (Ketogenic Diet)	Inhibit HDAC	Promotion of nuclear factor erythroid 2-related factor 2/antioxidant response element (Nrf2/ARE) pathway activation [223].
Antioxidant Stress	D4-Lnn	Activate Nrf2/ARE pathway	Reduction of excessive ROS generation, enhancement of protective gene expression, and inhibition of damaging protein production [190].
Antioxidant Stress, Mitochondrial Protection	Edaravone	Inhibit 5-LOX	Inhibition of 5-LOX activation, maintenance of mitochondrial ultrastructure and functional integrity, thereby protecting neurons from ischemic injury [216].
Reduce Mitochondrial Damage	Hydrogen	Scavenge ROS	Reduction of oxidative stress and protection of neural cells from ROS damage [167].
Reduce Mitochondrial Damage	Autotaxin Inhibitors (HA130 or PF8380) or Autotaxin/LPA Receptor Inhibitor (BrP-LPA)	Inhibit the autotaxin signaling pathway	Inhibition of Lysophosphatidic Acid (LPA)-mediated endothelial permeability [224].
Reduce Mitochondrial Damage	Triheptanoin	Against Long-Chain Fatty Acid Oxidation Disorders (LC-FAOD)	Generation of succinate to improve mitochondrial function and maintain cellular energy status [163].
Reduce Mitochondrial Damage, Neurorepair	Single Long-chain ω-3 FA	Modulate lipid metabolism and inflammation	Modulation of lipid metabolism [129], inflammatory responses [207], provision of neuroprotection, and promotion of neural repair and regeneration [214].
Reduce Mitochondrial Damage	Arachidonyl-2-Chloroethylamide (ACEA)	Activate CB1R	Improvement of mitochondrial function, thereby reducing GSK-3β phosphorylation [225].
Antioxidant Stress, Reduce Mitochondrial Damage	2-Arachidonoylglycerol (2-AG) and Anandamide (AEA)	-	Maintenance of mitochondrial function, reduction of calcium influx, and ROS generation [166]
Neuroprotection	Trans and Cis-Hinokiresinol	Antagonize CB1R, CB2R	Inhibition of 2-Arachidonoylglycerol (2-AG)-induced microglial migration [226].
Neurogenesis	PGE1	Regulate HIF-1α pathway	Promotion of endogenous NSCs proliferation and migration [213, 227].
Cholesterol Lowering, Mitochondrial Protection	Atorvastatin	Inhibit HMG-CoA reductase	Reduction of oxidative stress, ROS production, neuronal apoptosis, and improvement of mitochondrial function [163]
Neuroprotection	VPA (2-n-Propylpentanoic acid)	Inhibit HDAC, induce Hsp70.1B	Inhibition of glial scar formation [191].
Anti-apoptosis, Anti-inflammatory	GW4869, Scyphostatin	Inhibit SMase	Regulation of Cer synthesis and reduction of Cer levels [153, 228].

## LIST OF ABBREVIATIONS

2-AG: 2-Arachidonoylglycerol
20-HETE: 20-Hydroxyeicosatetraenoic Acid
4-HHE: 4-Hydroxyhexenal
4-HNE: 4-Hydroxy-2-Nonenal
AA: Arachidonic Acid
ACEA: Arachidonyl-2-chloroethylamide
AEA: Anandamide
ASK1: Apoptosis Signal-regulating Kinase 1
BBB: Blood-Brain Barrier
BCCAO: Bilateral Common Carotid Artery Occlusion
BHB: β-Hydroxybutyrate
CB1: Type 1 Cannabinoid Receptor
Cer: Ceramide
CNS: Central Nervous System
COX: Cyclooxygenase
COX-2: Cyclooxygenase-2
CSO: Cottonseed Oil
CYP4A: Cytochrome P450 4A
D4-Lnn: Deuterated Linoleic Acid Polyunsaturated Fatty Acids
DAM: Disease-associated Microglia
DG: Diacylglycerol
DG-AA: Diacylglycerol-arachidonic Acid
DG-SA: Diacylglycerol-stearic Acid
DHA: Docosahexaenoic Acid
EPA: Eicosapentaenoic Acid
FAs: Fatty Acids
FFAs: Free Fatty Acids
GTA: Glycerol Triacetate
HDAC: Histone Deacetylases
HSPs: Heat Shock Proteins
IL-1β: Interleukin-1β
JNK: Jun N-terminal Kinase
Keap1: Kelch-like ECH-associated Protein 1
Keap1-Nrf2: Kelch-like ECH-associated Protein 1-Nuclear Factor Erythroid 2-related Factor 2
LARAs: Lipid-associated Reactive Astrocytes
LC-FAOD: Long-chain Fatty Acid Oxidation Disorders
LCACs: Long-chain Acylcarnitines
LDRMs: Lipid Droplet-associated Microglia
LDs: Lipid Droplets
LOX: Lipoxygenase
LPC: Lysophosphatidylcholine
LPLs: Lysophospholipids
LTs: Leukotrienes
LXA4: Lipoxin A4
MaR: Maresins
MCAO: Middle Cerebral Artery Occlusion
MDA: Malondialdehyde
MMP: Matrix Metalloproteinase
MRI: Magnetic Resonance Imaging
NF-κB: Nuclear Factor-κB
NMDA: N-methyl-D-aspartate Receptors
NO: Nitric Oxide
NPD1: Neuroprotectin D1
Nrf: Nuclear Factor Erythroid 2-related Factor
Nrf2/ARE: Nuclear Factor Erythroid 2-related Factor 2/Antioxidant Response Element
NSAIDs: Non-steroidal Anti-inflammatory Drugs
NSCs: Neural Stem Cells
OGD: Oxygen-glucose Deprivation
OPCs: Oligodendrocyte Precursor Cells
PAF: Platelet-activating Factor
PC: Phosphatidylcholine
PD1: Protectin D1
PE: Phosphatidyl Ethanolamine
PGD2: Prostaglandin D2
PGE2: Prostaglandin E2
PIP: Phosphatidylinositol 4-phosphate
PIP2: Phosphatidylinositol 4,5-bisphosphate
PIPs: Polyphosphoinositides
PLA2: Phospholipase A2
PLC: Phospholipase C
PLIN2: Fatty Acid-binding Protein 2
pMCAO: Permanent Middle Cerebral Artery Occlusion
PP2A: Protein Phosphatase 2A
PPARs: Peroxisome Proliferator-activated Receptors
PS: Phosphatidylserine
PUFAs: Polyunsaturated Fatty Acids
ROS: Reactive Oxygen Species
RVs: Resolvins
S1P: Sphingosine-1-phosphate
S1PR: Sphingosine-1-phosphate receptors
SAMC: Stroke-associated Myeloid Cell
SCFAs: Short-chain Fatty Acids
SM: Sphingomyelin
SMase: Sphingomyelinase
SPMs: Specialized Pro-resolving Mediators
SVZ: Subventricular Zone
TG: Triacylglycerol
TIA: Transient Ischemic Attack
tMCAO: transient Middle Cerebral Artery Occlusion
TNF-α: Tumor Necrosis Factor-α
TXA2: Thromboxane A2
TXB2: Thromboxane B2
UCCAO: Unilateral Common Carotid Artery Occlusion
UCPs: Uncoupling Proteins
VPA: Valproic Acid
